# A Predictive Model for Prognosis and Therapeutic Response in Hepatocellular Carcinoma Based on a Panel of Three MED8-Related Immunomodulators

**DOI:** 10.3389/fonc.2022.868411

**Published:** 2022-04-26

**Authors:** Xiaojun Jin, Yongfei Song, Zhanglu An, Shanshan Wu, Dihui Cai, Yin Fu, Chuanjing Zhang, Lichao Chen, Wen Tang, Zequn Zheng, Hongsheng Lu, Jiangfang Lian

**Affiliations:** ^1^ School of Medicine, Ningbo University, Ningbo, China; ^2^ Department of Cardiovasology, The Affiliated Lihuili Hospital, Ningbo University, Ningbo, China; ^3^ Central Laboratory, Ningbo Institute of Innovation for Combined Medicine and Engineering, Ningbo, China; ^4^ Graduate School, Hebei North University, Zhangjiakou, China; ^5^ Department of Pathology, Taizhou Central Hospital (Taizhou University Affiliated Hospital), Taizhou, China

**Keywords:** MED8, HCC, immunomodulator, prognosis, immunotherapy, drug therapy

## Abstract

The current tumor-node-metastasis (TNM) system is limited in predicting the survival and guiding the treatment of hepatocellular carcinoma (HCC) patients since the TNM system only focuses on the anatomical factors, regardless of the intratumoral molecule heterogeneity. Besides, the landscape of intratumoral immune genes has emerged as a prognostic indicator. The mediator complex subunit 8 (MED8) is a major polymerase regulator and has been described as an oncogene in renal cell carcinoma, but its pathophysiological significance of HCC and its contribution to the prognosis of HCC remain unclear. Here, we aimed to discuss the expression profile and clinical correlation of MED8 in HCC and construct a predictive model based on MED8-related immunomodulators as a supplement to the TNM system. According to our analyses, MED8 was overexpressed in HCC tissues and increased expression of MED8 was an indicator of poor outcome in HCC. The knockdown of MED8 weakened the proliferation, colony forming, and migration of HepG2 and Huh7 cells. Subsequently, a predictive model was identified based on a panel of three MED8-related immunomodulators using The Cancer Genome Atlas (TCGA) database and further validated in International Cancer Genome Consortium (ICGC) database. The combination of the predictive model and the TNM system could improve the performance in predicting the survival of HCC patients. High-risk patients had poor overall survival in TCGA and ICGC databases, as well as in subgroup analysis with early clinicopathology classification. It was also found that high-risk patients had a higher probability of recurrence in TCGA cohort. Furthermore, low-risk score indicated a better response to immunotherapy and drug therapy. This predictive model can be served as a supplement to the TNM system and may have implications in prognosis stratification and therapeutic guidance for HCC.

## Introduction

Hepatocellular carcinoma (HCC) is a predominant histological form of liver cancer with dismal long-term prognosis and high recurrence rate (1). Surgical resection may significantly benefit early-stage patients. However, several HCC patients are first diagnosed at advanced stages and present local metastasis wherein the surgical excision treatment is unsuitable. The median survival for patients with advanced HCC is less than a year (2). Given the grim status of HCC, novel and effective treatment strategies are required. Immune checkpoint blockade (ICB)-based immunotherapy has brought a paradigm shift in cancer treatment (3). Recent data from phase I/II clinical trial demonstrate that the combined administration of Opdivo (programmed death 1 inhibitor) and Yervoy (cytotoxic T lymphocyte-associated antigen-4 inhibitor) is promising for the treatment of HCC patients (4). However, only a subset of patients responds favorably to ICB therapy due to the heterogeneity and complex molecular pathogenesis underlying HCC. Cancer cells can suppress the function of immune effector cells changing the immune molecular expression and immune cell aggregation in the intratumoral microenvironment, therapy resulting in immune escape (5). Furthermore, the engulfment of the tumor in this intratumor immune landscape can change its response to adjuvant therapy and impact the progression of malignancy (6). Understanding the regulatory machinery of HCC immunology and identifying immune-related gene signatures are warranted for predicting the therapeutic sensitivity to different drugs.

The prediction of survival and recurrence in HCC patients and treatment decisions are primarily dictated by the tumor-node-metastasis (TNM) classification system based on the tumor size, nodal involvement, and distant metastasis. However, owing to the molecular heterogeneity in HCC, determining the alterations in predictive indicator landscapes for the prognosis of HCC are underway (7, 8). Changes in cancer cells including their molecular biology and genomics are responsible for the progression of the disease and in the past few years, have been used to stratify HCC patients into specific prognosis groups (9). The TNM classification system relies only on the anatomic factors regardless of the intertumoral molecule heterogeneity; and it fails to consider the personal prognoses of each patient. Furthermore, the landscape of intratumoral immune genes has recently emerged as a prognostic indicator to assist in clinical decision-making (6). Therefore, constructing an immune-related model not only aids the prediction of therapeutic sensitivity but also compensates for the unsatisfactory capacity of survival and recurrence predictions in the TNM system. While several immune-related gene models have been constructed for HCC, most of them cannot concurrently predict the effects on survival and therapeutic responses; moreover, their long-term prediction power for survival is unsatisfactory (7, 10, 11). Besides, few of them assessed the predictive power of models for recurrence in HCC patients.

Mediator, a multiprotein complex, is required for the mRNA transcriptional process. It acts as a bridge between RNA polymerase II and regulatory proteins (12). The mediator complex subunit 8 (MED8) is located at the head module of mediator and is a major polymerase regulator (13). MED8 has been implicated in the development of clear cell renal cell carcinomas (ccRCC) (14). A previous study reports that MED8 is involved in inhibiting the interferon (IFN) responses in human lung adenocarcinoma cells (15). This finding implies potential functional links between MED8 and tumor immunity. Furthermore, the pathophysiological significance of MED8 in HCC and its contribution to the prognosis of HCC remain unclear. In this study, we assessed the expression and prognostic value of MED8 in HCC patients and unveiled potential roles of MED8 underlying HCC tumor progression by generation of small interfering RNA (siRNA)-mediated MED8 knockdown HCC cell lines; we further explored whether a correlation existed between MED8 and HCC immune through bioinformatics analysis. Since MED8 expression could influence the proportion of tumor-infiltrating immune cells in HCC by Estimating Relative Subsets of RNA Transcripts (CIBERSORT) analysis; the MED8-related immunomodulators were subsequently identified from the TISIDB website. Finally, based on a panel of three MED8-related immunomodulators, a predictive model for prognoses and therapeutic responses in HCC was constructed.

## Methods

### Data Mining in The Cancer Genome Atlas and International Cancer Genome Consortium Databases

The transcriptomic data and corresponding clinical information of HCC cases were acquired from TCGA liver hepatocyte carcinoma (TCGA-LIHC) project till April 26, 2021, and were used as the training set. It included 365 tumorous specimens and 50 adjacent non-tumor tissues (**Supplementary Table 1**). The transcriptomic data and clinical information of 240 HCC cases were obtained from the International Cancer Genome Consortium (ICGC) portal (LIRI-JP project, https://dcc.icgc.org), and was used as a validation set (**Supplementary Table 1**). The “limma” package in R was used to analyze the mRNA expression profile of MED8 in tumor and adjacent non-tumor tissues in TCGA-LIHC cohort. The expression of MED8 among 50 paired tumor tissue and the corresponding adjacent non-tumor specimens was compared. The correlation of MED8 expression with the clinicopathological features was explored. TCGA-LIHC cohort cases were stratified into low- or high- groups based on the median expression of MED8, the difference in the overall survival (OS) between the groups was analyzed. Univariate and multivariate Cox proportional hazards regression analyses were used to identify the independent factors that affecting OS in TCGA-LIHC patients.

### Gene Set Enrichment Analysis and CIBERSORT Analysis

TCGA-LIHC cohort was stratified into high- or low- groups based on the median mRNA expression of MED8. The potential pathways upregulated due to high MED8 expression were identified by GSEA. The annotated gene sets from c2.cp.kegg.v7.4.symbols.gmt were used as the reference. The number of gene set permutations was selected as 1,000 in each analysis. The significant pathways were filtered by the normalized enrichment scores (NES) > 1, false discovery rate < 0.25 and nominal *p* < 0.05. The CIBERSORT analysis is a deconvolution algorithm that predicts the 22 immune cell type proportions from gene expression signature (16). The proportions of tumor-infiltrating immune cells in high- and low- groups was inferred by the CIBERSORT online tool (https://cibersort.stanford.edu/runcibersort.php), followed by filtration with the criterion of *P* < 0.05. The correlation between the level of MED8 expression and the proportion of tumor-infiltrating immune cells was examined using the Spearman correlation test.

### Construction of the Predictive Model Based on MED8-Related Immunomodulators

The candidate MED8-related immunomodulators including immunoinhibitors and immunostimulators were retrieved from TISIDB (http://cis.hku.hk/TISIDB/index.php), a website for predicting immune-tumor interactions (17). The inclusion criteria for the significantly related immunomodulators were based on the Spearman correlation results and *P*-value < 0.05. First, the univariate analysis was used along with the Cox proportional hazards regression model to identify significant immunomodulators that affected the OS of patients in TCGA-LIHC cohort. The resulting immunomodulators filtered using univariate analysis were used as the input for multivariate Cox analysis. Thus, a predictive model was constructed and the stepwise variables were selected with the Akaike information criteria executed using the “stepAIC” algorithm in the “MASS” package of R (18). The final prediction model was built based on the optimal variables. Risk scores considered as the prognostic index and were calculated from the equation: risk score = gene 1 expression × gene 1 coefficient + gene 2 expression × gene 2 coefficient + … + gene *n* expression × gene *n* coefficient. The coefficient of each gene was obtained from the Cox model. The cut-off for the risk scores came from the predictive model that separated the cases into low-risk and high-risk groups and was identified in X-tile software (version 3.6.1).

### Evaluation of the Predictive Model in Prognosis

The clustering abilities of the risk scores derived from TCGA-LIHC cohort were visualized using the t-distributed stochastic neighbor embedding (t-SNE) algorithm and principal component analysis. Survival differences between low-risk and high-risk groups were assessed using Kaplan-Meier curves and log-rank test. Stratified OS analyses were conducted to assess the differences in the subgroups of early clinicopathology classifications including T1 stage, N0 stage, M0 stage, pathological stage 1, and histological grade 1. The prediction accuracy of the predictive model in 1-year, 3-year, and 5-year OS was assessed and compared with the TNM model that incorporated stage and grade by measuring the area under the curve (AUC) of the receiver operating characteristic (ROC) curve *via* the “timeROC” package of R. ROC curve analyses were used to determine whether our model had better prediction accuracy relative to the previously published immune-related models for prediction of long-term survival. The “Meng model”, “Dai model”, and “Wang model” were included in the comparison, which were reported to have a good performance in prognostic prediction (7, 10, 11).

A validation set from the ICGC cohort was adopted to externally validate the prognostic performance of the model. The risk scores were figured for each patient in the ICGC cohort with the same formula, and the patients were then categorized into low-risk and high-risk groups using the same cut-off. KM curve, ROC, PCA, and t-SNE analyses was performed as described above. ROC analyses were only conducted for 1-year and 3-year OS due to the small number of cases with the OS time of more than 5-year in the ICGC cohort.

### Validation of Predictive Values of the Model for HCC Recurrence

To verify the predictive value of the model for recurrence of HCC, the TCGA-LIHC cohort specimens were classified into low-risk and high-risk groups in line with the above cut-off. Significant differences for comparing the disease-free survival (DFS) time were assessed with by the KM curve analysis. The performance of the predictive model in DFS was estimated using ROC analysis as described above.

### Construction and Verification of the Nomogram

For quantifying the survival probability for each patient in TCGA-LIHC cohort, the nomogram was developed based on TCGA-LIHC cohort by incorporating the clinicopathological features and risk scores of patients using the R “rms” package. Plotting a calibration described the consistency between the predicted and actual probabilities of the OS through the bootstrap method with 1,000 replicates.

### Prediction of the Therapeutic Responses in Patients Having Different Risk Scores

The power of the predictive model in predicting ICB therapeutic responses in HCC patients was inferred from the immunophenoscore (IPS) derived from the Cancer Immunome Atlas (https://tcia.at/home) and the tumor immune dysfunction and exclusion (TIDE) score derived from the TIDE online website (http://tide.dfci.harvard.edu/). The IPS value positively reflected the tumor immunity from the expression of crucial components of tumor immunogenicity; the TIDE prediction scores represented the potential for tumor immune escape. The lower the TIDE score and the higher the IPS value, the better the therapeutic response to ICB. Single sample gene set enrichment analysis (ssGSEA) was used to examine the diversities in the activities of 13 immune-related pathways and infiltration scores for 16 immune cells types between the high-risk and low-risk groups of TCGA-LIHC cohort using the R “GSVA” package. The predictive capacity of the model for response to chemotherapeutic and targeted drugs was evaluated using R “pRRophetic” package and the Genomics of Drug Sensitivity in Cancer web tool (https://www.cancerrxgene.org/) (19). The half-maximal inhibitory concentration (IC50) represented the chemotherapeutic sensitivity in the different risk groups of TCGA-LIHC. To examine the differences in the biological pathways between the low-risk and high-risk groups of TCGA-LIHC cohort, GSEA was conducted as described above.

Furthermore, the ICGC cohort was also adopted to externally verify the predictive capacity of the model in responding therapeutic sensitivity. The ICB therapy response of low-risk and high-risk groups of ICGC cohort was only inferred by the TIDE value due to the restricted dataset of the Cancer Immunome Atlas.

### Immunohistochemistry

The prepared paraffin-embedded sections of HCC and non-tumor liver tissues were sequentially deparaffinized, rehydrated, and antigen retrieval was performed by by heating treatment in citrate buffer. After blocking with 3% bovine serum albumin, the sections were stained with the primary antibody, and polyclonal rabbit anti-MED8 (1:200, Affinity), and kept overnight at 4°C. The following day after the primary antibody incubation, the sections were washed and stained with horseradish peroxidase-conjugated secondary antibody (1:200, Abcam) for one hour at 37°C and visualized using diaminobenzidine.

### Cell Culture and Transfection

Human HCC cell lines, HepG2, and Huh7 were obtained from the cell bank of the Chinese Academy of Science (Shanghai, China). For the generation of HepG2 with MED8 knocked down (HepG2 KD) and Huh7 with MED8 knocked down (Huh7 KD), cells were transfected with 50nM specific siRNA (Gene-Pharma, China) constructs for human MED8 sequence using Lipofectamine 2000 (Invitrogen, USA). The scrambled siRNA construct was used as the negative control (NC). Cells were cultured in Dulbecco’s modified eagle medium supplemented with 10% fetal bovine serum (37°C and 5% CO_2_) and transfected when the confluency reached 50%. The specific siRNA constructs for MED8 are listed in **Supplementary Table 2**.

### RNA Extraction and Quantitative Real-Time Polymerase Chain Reaction

Total RNA from cells was isolated by the TRIzol method (Invitrogen, USA) and dissolved in 50μl RNase-free water. The RNA (1 ug) was reverse-transcribed using the reverse transcription kit (Takara, Japan). The qRT-PCR reaction was performed using the SYBR Premix Ex Taq kit (Takara, Japan) according to the manufacturer’s instructions. The relative differences in mRNA expression were measured by the 2^−ΔΔCT^ method. Primer sequences of the genes adopted for qRT-PCR are specified in **Supplementary Table 3**.

### Western Blotting

The total protein quantity from HCC cells was determined by the bicinchoninic acid assay. The proteins were separated on sodium dodecyl sulfate gel and transferred onto the PVDF membranes. Proteins on membranes were blocked with 5% non-fat milk for 2 hours, probed with primary antibodies (1: 1000) overnight at 4°C and thereafter incubated with the secondary antibody (1: 10000) for 1 hour. The enhanced chemiluminescence (Beyotime) was used to visualize the brands. The antibodies used are specified in **Supplementary Table 4**.

### Cell Proliferation and Clonogenic Assays

Cells of HepG2 KD, HepG2 NC, Huh7 KD and Huh7 NC groups were harvested and seeded in 96-well plates at a density of 2,000 cells per well. The 10μl of cell counting kit-8 solution (Yeasen) was added to the cells at 12, 24, 36, 48, 72, and 96 hours after seeding before measuring the absorbance at 450nm. For the clonogenic assay, cells of each group were seeded in six-well plates at 1,000 cells per well. After incubation for two weeks, cells were fixed in 4% paraformaldehyde for 30 minutes, stained with 0.1% crystal violet solution, and counted.

### Migration Assay

Cell migration abilities of cells treated with specific siRNA or scramble siRNA constructs were assessed by the Transwell chambers (Corning) assays. A total of 1 × 10^5 cells were seeded onto the upper chamber. After incubation for 48 hours, the migrated cells were fixed in 4% paraformaldehyde and stained with crystal violet solution.

### Statistical Analysis

All data analyses were conducted using the R software (Version 4.0.3) and Prism software (GraphPad software Inc., version 9.0.0). The data are presented as mean ± standard deviation. *In vitro* assays were independently repeated a minimum of three times. Pairwise comparisons were performed using Student’s *t*-test for normally distributed variables or the Wilcoxon rank-sum test for non-normally distributed variables. *P*-values < 0.05 were considered statistically significant.

## Results

### Expression and Prognostic Roles of MED8

**Figure 1:**
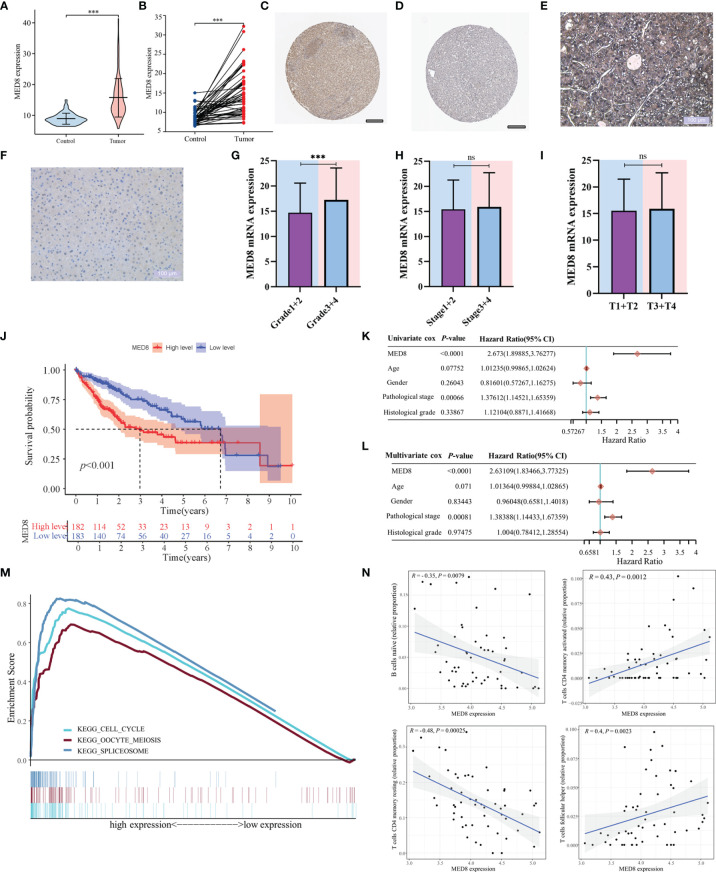
The correlation of MED8 expression with prognoses, pathways, and immune infiltration in HCC. **(A)** The levels of MED8 mRNA expression in tumor and non-tumorous liver tissues from TCGA cohort. **(B)** The levels of MED8 mRNA expression in 50 matched tumor and non-tumorous liver tissues. **(C, D)** The levels of MED8 protein expression in tumor **(C)** and non-tumor liver tissues **(D)** from HPA database, scar bar: 200μm. **(E, F)** The immunohistochemistry staining of tumor **(E)** and non-tumor liver tissues **(F)**, scar bar: 100μm. **(G–I)** The levels of MED8 mRNA expression in HCC patients with different grade **(G)**, stage **(H)** and T stage **(I)** from TCGA-LIHC cohort. **(J)** The comparison of overall survival between high MED8 expression and low MED8 expression groups from TCGA-LIHC cohort. **(K, L)** The univariate **(K)** and multivariate Cox regression **(L)** analyses. **(M)** The pathways from GSEA analysis. **(N)** Correlation between MED8 expression and tumor-infiltrating immune cells. ***: *P* < 0.001; ns, no significance.

The mRNA expression of MED8 was significantly elevated in TCGA-LIHC tissues relative to the adjacent non-tumor liver tissues (**Figures 1A, B**). According to the HPA database (**Figures 1C, D**) and IHC staining (**Figures 1E, F**), the MED8 protein expression was enhanced in HCC. Additionally, the high expression of MED8 was associated with poor OS (**Figure 1J**) and the advanced histological grade (**Figures 1G** **–I**) of TCGA-LIHC cohort. Univariate and multivariate identified high expression of MED8 as a detrimental independent prognostic factor for OS in HCC patients (**Figures 1K, L**).

### Functional Enrichment and CIBERSORT Analyses

In the aforementioned results, high MED8 expression was significantly correlated with poor prognosis and advanced TNM parameters in HCC. GSEA was used to examine the pathways upregulated due to high MED8 expression in HCC. The results of GSEA showed that the top three enrichment pathways according to the NES scores were in the processes of the cell cycle, oocyte meiosis, and spliceosome (**Figure 1M**). The results of CIBERSORT analysis showed that proportions of naïve B cells and resting memory CD4+ T cells was negatively correlated with MED8 expression and those of activated memory CD4+ T cells and follicular helper T cells exhibited positive correlations in HCC (**Figure 1N**).

### Evaluation of the Oncogenic Effect of MED8 on HCC Cells

**Figure 2:**
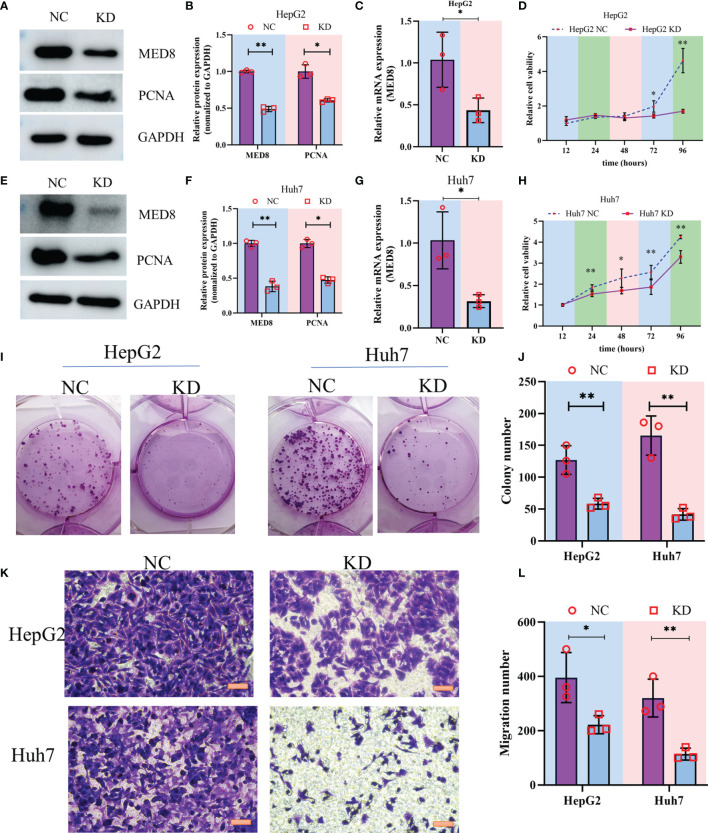
The impact of MED8 knockdown in HCC cell lines. **(A, E)** Western blot analysis of the MED8 and PCNA expression levels in HepG2 **(A)** and Huh7 cells **(E)** after MED8 knockdown. **(B, F)** Relative quantitative analysis of MED8 protein expression level in HepG2 **(B)** and Huh7 cells **(F)** after MED8 knockdown. **(C, G)** qRT-PCR analysis of the MED8 expression level in HepG2 **(C)** and Huh7 cells **(G)** after MED8 knockdown. **(D, H)** The proliferation ratio of HepG2 **(D)** and Huh7 cells **(H)** from CCK-8 assays. **(I, J)** Colony-forming assays of HepG2 and Huh7 cells. **(K, L)** Migration assays of HepG2 and Huh7 cells. Scar bar: 50μm. *: *P* < 0.05; **: *P* < 0.01.

To characterize the pathophysiological significance of MED8 in HCC cell lines, both mRNA and protein expressions of MED8 were suppressed in HepG2 cells (**Figures 2A** **–C**) and Huh7 cells (**Figures 2E** **–G**) using siRNA constructs. Proliferating cell nuclear antigen (PCNA), a cell proliferation marker, reflected the status of cell proliferation. The protein level of PCNA reduced significantly after siRNA treatment. The results of CCK-8 assay indicated that the MED8 knockdown inhibited the proliferation rates in HepG2 cells and Huh7 cells compared to their respective controls (**Figures 2D, H**). Furthermore, siRNA-mediated MED8 knockdown could significantly inhibit the clonogenicity (**Figures 2I, J**) and migration (**Figures 2K, L**) in HepG2 cells and Huh7 cells. These results indicated that MED8, indeed, played an essential role in the maintenance of proliferation and migration in the HCC cells, which suggested its potential as a promising target for HCC treatment.

### Construction of a Predictive Model and Validation of Its Prognostic Value

**Figure 3:**
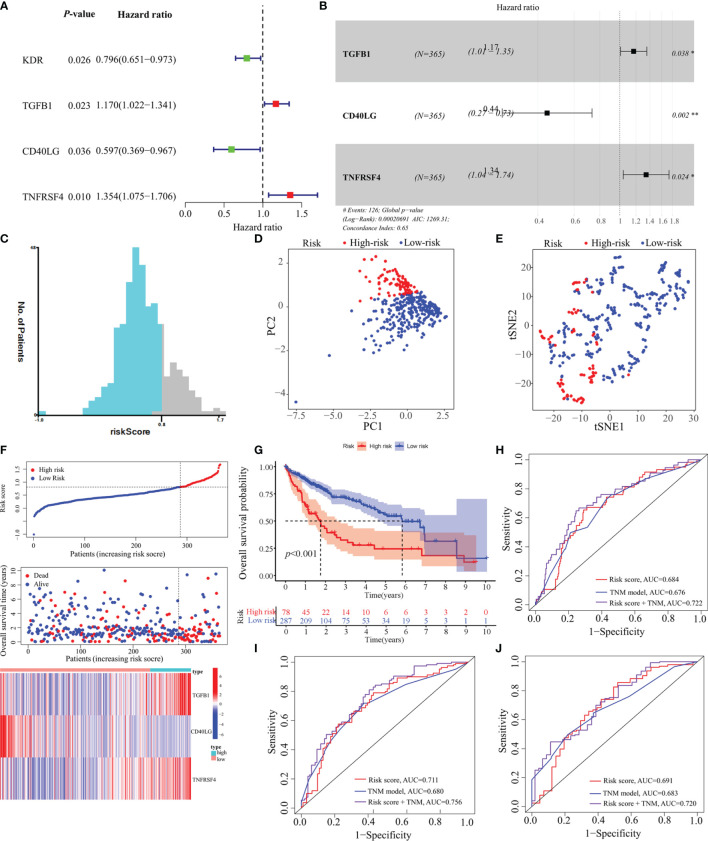
Construction of a predictive model based on MED8-related immunomodulators. **(A)** Univariate Cox regression analysis of 25 immunomodulators. **(B)** Forest plot exhibiting the multivariate Cox regression results. **(C)** The optimal cut-off of predictive model. **(D)** PCA analysis of TCGA-LIHC cohort. **(E)** t-SNE analysis of TCGA-LIHC cohort. **(F)** Risk score, survival status and expression of MED8-related immunomodulators in high-risk and low-risk groups of TCGA-LIHC cohort. **(G)** Overall survival analysis in high-risk and low-risk groups of TCGA-LIHC cohort. **(H–J)** ROC analyses of 1- **(H)**, 3- **(I)** and 5-years **(J)** overall survival in TCGA-LIHC cohort. *: *P* < 0.05; **: *P* < 0.01.

In total, 18 immunostimulators (**Supplementary Figure S1**) and 7 immunoinhibitors (**Supplementary Figure 2**) were found to be significantly associated with MED8 expression in HCC. In TCGA-LIHC database, using univariate analysis, we identified four immunomodulators as the significant prognostic variables for OS in HCC (**Figure 3A**). These variables were included in the stepwise multivariate Cox regression analysis, which was further used to generate a predictive model based on TGFB1, CD40LG, and TNFRSF4 (**Figure 3B**). The risk scores were calculated using the following equation: risk scores = TGFB1 × 0.155 – CD40LG × 0.8123 + TNFRSF4 × 0.295. TCGA-LIHC cohort was divided into low-risk and high-risk groups according to the optimal cut-off (0.810) obtained from the X-tile software (**Figures 3C, F**). The results of PCA and t-SNE analyses showed that low-risk and high-risk patients were distributed in two discrete groups (**Figures 3D, E**).

**Figure 4:**
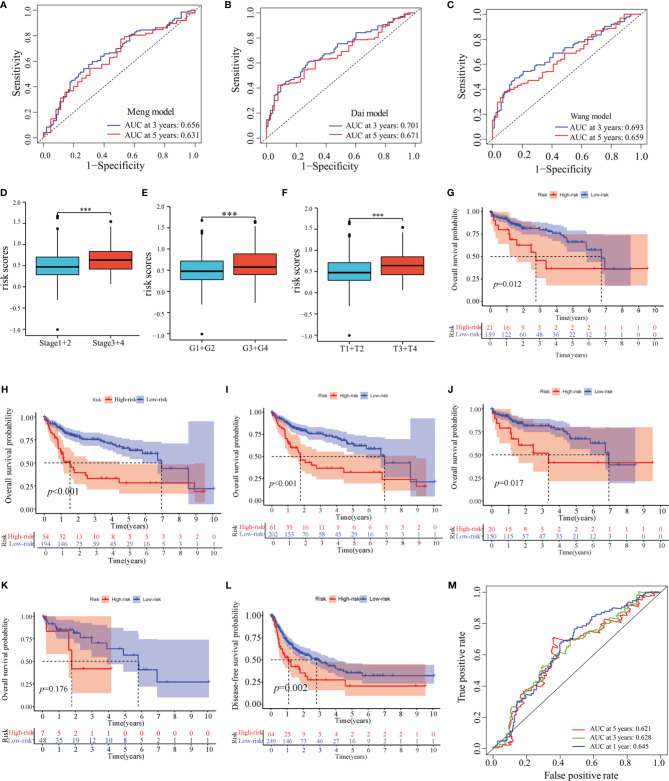
Prognostic values of the predictive model in TCGA-LIHC cohort. **(A–C)** ROC analyses of the other published immune-related models. **(D–F)** Distribution of risk scores in pathological stage **(D)**, histological grade **(E)** and T stage **(F)** of TCGA-LIHC cohort. **(G–K)** Overall survival analyses between low-risk and high-risk subgroups in T1 stage **(G)**, N0 stage **(H)**, M0 stage **(I)**, pathological stage 1 **(J)** and histological grade 1 **(K)**. **(L)** Disease-free survival analysis in high-risk and low-risk groups of TCGA-LIHC cohort. **(M)** ROC analyses of disease-free survival in TCGA-LIHC cohort. ***: *P* < 0.001.

In TCGA-LIHC cohort, the KM curve analysis showed that patients in the high-risk group had a worse OS than those in the low-risk group (**Figure 3G**). The AUCs of the model were 0.684, 0.711, and 0.691 for 1-, 3- and 5-years OS curves, respectively, which was better than the TNM model, indicating the predictive model had a considerably good value in predicting the OS of HCC patients (**Figures 3H–J**). ROC analyses, along with the risk scores and the TNM model, increased the AUCs to 0.722, 0.756, and 0.720 for 1-, 3- and 5-years of OS, respectively. Moreover, our model showed superior accuracy in predicting long-term survival including 3- and 5-year OS relative to the previously published immune-related models (**Figures 4A** **–C**). There was a positive correlation between risk scores and multiple clinicopathological features (**Figures 4D** **–F**). The stratified survival analyses revealed that patients in the high-risk subgroup had poor OS in T1 stage (**Figure 4G**), N0 stage (**Figure 4H**), M0 stage (**Figure 4I**) and pathological stage 1 (**Figure 4J**). No significance was observed in histological grade 1, probably due to the small amount (**Figure 4K**).

Next, we determined whether this model could be used to predict the DFS. The KM curve analysis showed that the DFS for patients with high-risk scores was shorter than for those with low-risk scores (**Figure 4L**). The AUCs of the model were 0.645, 0.628, and 0.621 for 1-, 3- and 5-years DFS, respectively. (**Figure 4M**).

**Figure 5:**
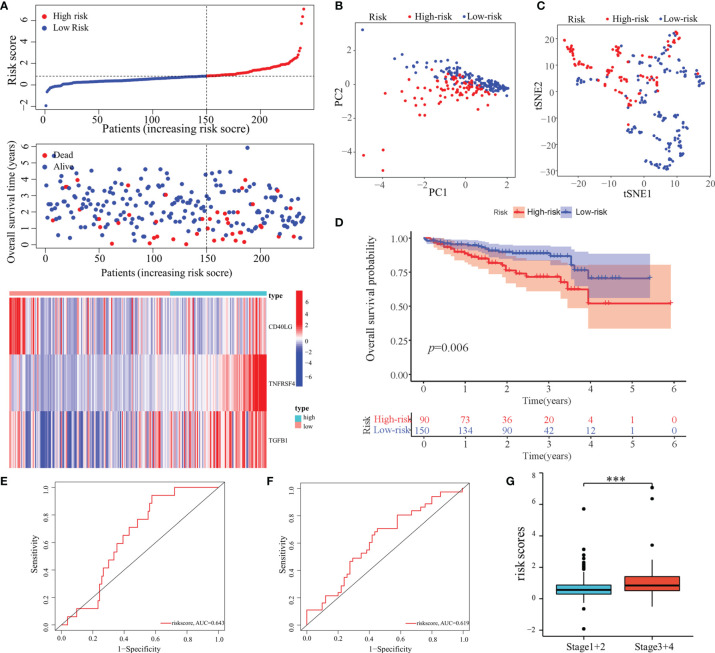
Validation of the predictive model in the ICGC cohort. **(A)** Risk score, survival status, and expression of MED8-related immunomodulators in high-risk and low-risk groups of ICGC cohort. **(B)** PCA analysis of ICGC cohort. **(C)** t-SNE analysis of ICGC cohort. **(D)** Overall survival analysis in high-risk and low-risk groups of ICGC cohort. **(E, F)** ROC analyses of 1- **(E)** and 3-years **(F)** overall survival in the ICGC cohort. **(G)** Distribution of risk scores in different pathological stage of the ICGC cohort. ***: *P* < 0.001.

Furthermore, the predictive performance of the model was validated in the ICGC cohort to validate its predictive performances. The risk scores, OS, and risk-gene expression in the ICGC cohort are presented in the dot plot (**Figure 5A**). Likewise, PCA and t-SNE analyses showed that the patients were bimodally distributed (**Figures 5B, C**). The KM curve analysis indicated high-risk patients had worse OS (**Figure 5D**). The AUCs were 0.643 and 0.619 for 1- and 3-years OS, respectively (**Figures 5E, F**). Similar to TCGA-LIHC cohort, a positive association of the risk scores with the pathological stage was found (**Figure 5G**).

### Construction and Verification of the Nomogram

**Figure 6:**
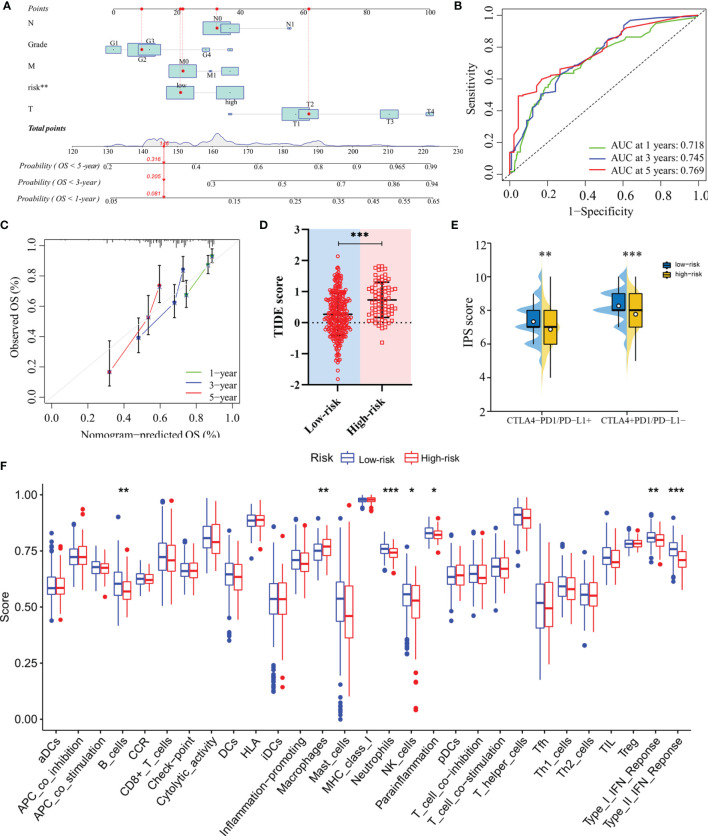
Construction of the nomogram and analysis of immunotherapeutic response. **(A)** The nomogram for predicting 1-, 3- and 5-year overall survival in TCGA-LIHC cohort. **(B)** ROC analyses of the nomogram predicting of 1-, 3- and 5-year overall survival. **(C)** Calibration curve for validation of the nomogram. **(D)** TIDE scores of high-risk and low-risk groups in TCGA-LIHC cohort. **(E)** IPS scores of high-risk and low-risk groups in TCGA-LIHC cohort. **(F)** Single-sample gene set enrichment analysis. *: *P* < 0.05; **: *P* < 0.01; ***: *P* < 0.001.

The nomogram, after integrating the risk scores and clinicopathological features for quantitatively predicting the probabilities of 1-, 3- and 5-years OS for individual patients, was constructed (**Figure 6A**). The AUCs were 0.718, 0.745, and 0.769 for predicting in 1-, 3- and 5-year OS, respectively (**Figure 6B**). The calibration curve exhibited a marked agreement between predicted and actual outcomes in the nomogram (**Figure 6C**).

### Evaluation of the Predictive Model in Therapeutic Response

**Figure 7:**
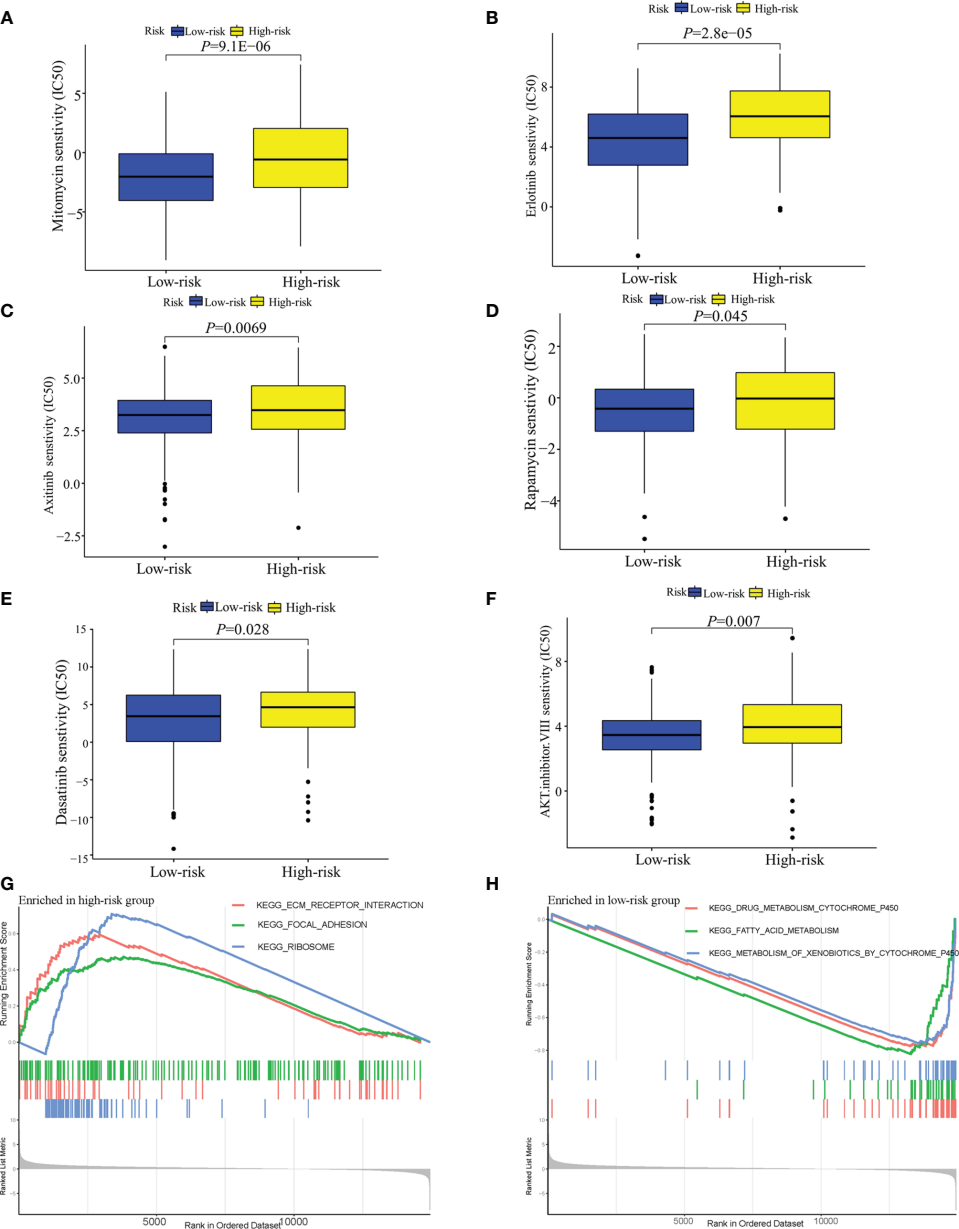
Analysis of the sensitivity toward drug therapy in high-risk and low-risk groups. **(A–F)** Sensitivity of high-risk and low-risk groups of TCGA-LIHC cohort to drug therapy. **(G)** The top three upregulated pathways in the high-risk group of TCGA-LIHC cohort. **(H)** the top three downregulated pathways in the low-risk group of TCGA-LIHC cohort.

**Figure 8:**
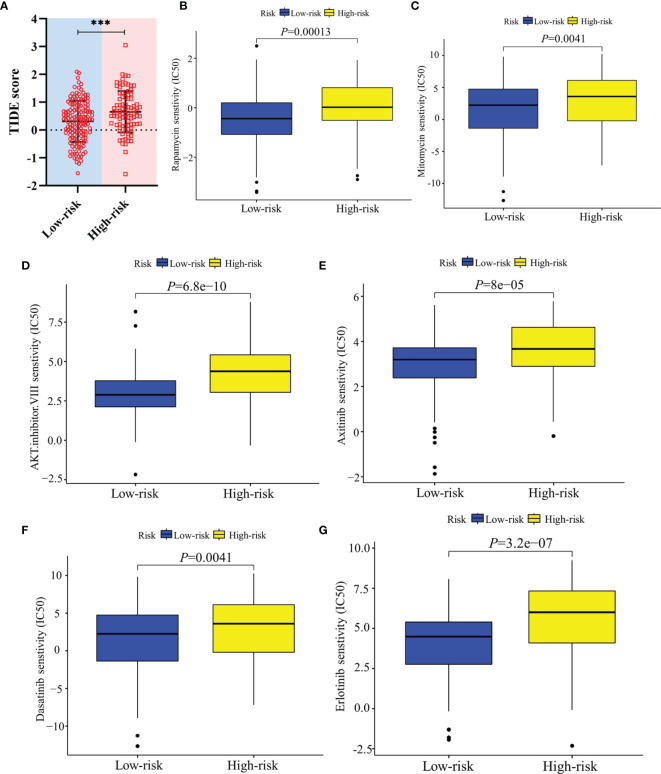
Validation of the therapeutic response in the ICGC cohort. **(A)** TIDE scores of high-risk and low-risk groups in the ICGC cohort. **(B–G)** Sensitivity of high-risk and low-risk groups of the ICGC cohort to drug therapy. ***: *P* < 0.001.

In terms of response to the ICB therapy, chemotherapy, and targeted therapy, patients in the low-risk group showed better therapy responses relative to those in the high-risk group (**Figures 6D, E**; **Figures 7A–F**), and similar results were found in the ICGC cohort (**Figures 8A–G**), which suggested that the predictive model had good power in discriminating the difference in therapeutic responses among the HCC patients. The scores for type- I IFN response, type-II IFN response, B cells, neutrophils and natural killer cells were lower in the high-risk group, while that for the macrophages showed an opposite trend (**Figure 6F**). Moreover, we performed GSEA to examine the differences in the signaling-pathways between the low-risk and high-risk groups; the top three enriched pathways based on the NES scores were displayed in each group (**Figures 7G, H**). The results showed that the signaling-pathways related to drug resistance, including the extracellular matrix (ECM) receptor interaction and focal adhesion, were significantly upregulated in the high-risk group.

## Discussion

The close relationship between tumorigenesis among multiple cancer types and numerous mediator complex subunits including MED1, MED12, MED16, and MED19 has been extensively reported (20–23). As for MED8, the correlation between MED8 expression and prognosis was only reported in ccRCC (14). In this study, MED8 was found to be upregulated in HCC, both at the mRNA and protein levels, and high MED8 expression was identified as an independent predictor of poor OS in HCC patients. The siRNA-mediated MED8 knockdown, *in vitro*, led to reduced proliferation and migration in both HepG2 and Huh7 cell lines, which confirmed the implication of upregulated MED8 implicated in development of HCC. Restricted cell proliferation and migration were reported after knocking down MED8 in the ccRCC cell line (14). In previous studies, knocking down oncogenes, MED23 and MED19, had been showed to inhibit growth and induce cell-cycle arrest in the HCC cell lines. Guo et al. reported that MED23 knockdown in HCC cells upregulated p16, which in turn arrested cell-cycle progression at the G1/S transition by inhibiting the activity of cyclin-dependent protein kinase, cyclin D1 complex (24). Zou et al. found that MED19 knockdown also led to cell-cycle arrest at G0/G1 phase in HCC cells. The MED8 knockdown may induce cell-cycle arrest to partially attenuate the growth in HCC cells (21). However, the mechanisms underlying the attenuated cell growth remain poorly understood after MED8 knockdown in HCC cells and further investigations are needed. Furthermore, KEGG enrichment analysis revealed that high MED8 expression upregulated the pathways involved in the cell cycle, oocyte meiosis, and spliceosome. The dysregulation of these pathways causes tumor progression in HCC, which could explain why the link of high MED8 expression was linked to advanced clinicopathological parameters (6). Using CIBERSORT analysis, we identified that MED8 expression was negatively correlated with the proportions of infiltrated resting memory CD4 T cells and naïve B cells and positively correlated with those of infiltrated active memory CD4+ T cells and follicular helper T cells, which suggested that the aberrant MED8 expression could alter the immune activity by influencing the tumor-infiltrating immune cells and MED8 was, indeed, linked to immunity in HCC.

Since MED8 was related to the prognosis and intratumoral immunity of HCC and several immune gene signatures have been previously reported to predict outcomes; we next evaluated whether the intratumoral MED8-related immune genes had the potential to generate a model with robust performance to guide therapy and supplement the prognosis prediction of TNM staging system. The MED8-related immunomodulators were extracted from the TISIDB website and used to construct a prediction model. Immunomodulators have implications in influencing the immune destruction and tumor escape (25). In this study, three immune genes were finally included in the prediction model. The adenovirus-mediated CD40LG gene therapy was reported to stimulate the antitumor effect in a rat model of HCC (26). It was reported that TGFB1 expression was upregulated in HCC cells and TGFB1-treated HCC cells accelerated cell migration and increased expression of N-cadherin (27). Furthermore, TNFRSF4 is a costimulatory molecule expressed in the regulatory T cells and its overexpression substantially correlates with high serum alpha-fetoprotein levels, vascular invasion, and poor survival in HCC patients (28). CD40LG was identified as a protective factor for survival, while TGFB1 and TNFRSF4 were the factors that adversely affected survival in HCC patients, which was in line with the aforementioned findings.

The present model had a robust performance in predicting prognosis, as it had been validated in the ICGC and TCGA databases. When compared with published immune-related models, the model reported in this study yielded superior performance in predicting long-term survival. Additionally, their models were constructed based on at least 10 variables, which was inconvenient for clinical application. The risk scores were positively associated with advanced clinicopathological parameters, which could in part explain the poor prognosis in patients with high-risk scores. The stratified survival analyses showed that patients in the high-risk subgroup had poor OS even in the early clinicopathological classification, which indicated that the predictive model could enable timely clinical intervention therapy, thereby improving the patients’ outcomes. Furthermore, we found that the patients with high-risk scores had poor DFS time and a favorable predictive performance for DFS. Although several models based on multiple gene panels have been reported for the prediction of OS and recurrence, these models only have the same variables when simultaneously predicting OS and recurrence; they do not consider the same risk threshold and coefficients, which reduces their clinical applicability (9, 29). Overall, these results confirmed that the predictive model based on MED8-related immunomodulators could serve as a supplement for the TNM staging system in predicting prognosis of HCC patients.

Since ICB therapy is an emerging option in the treatment of HCC and chemotherapy and targeted therapy are the first-line treatment strategies, we next predicted the response of HCC patients to ICB therapy and commonly used drugs in chemotherapy and targeted therapy using our model. Results of the TIDE and IPS analyses suggested that the low-risk group may more likely benefit from ICB therapy. Based on the ssGSEA analysis, patients in the high-risk group tended to exhibit immunosuppressed status, low immunity activity (including those of type-I IFN and type-II IFN responses), lower fractions of B cells, neutrophils, and natural killer cells. Type-I IFN has immunoregulatory functions, such as immunosurveillance pf tumor lesions; which exerts immunosuppressive effects against tumor progression (30). Suppression of type- I IFN signaling in tumors was reported to mediate resistance to ICB treatment (31). The tumor-infiltrating regulatory T cells (Tregs) permit tumor growth and neuropilin‐1 and is needed to maintain the stability and function of the intratumoral Tregs (32). Type-II IFN can promote tumor response to ICB immunotherapy by inducing the generation of intratumoral neuropilin-1-deficient Tregs (32). However, type-II IFN also enhances the expression of the programmed cell death protein 1-ligand 1 in HCC cells by regulation of the IFN regulatory factor 1 and, in turn, leads to immune escape of HCC (33). Therefore, the results showing that low-risk patients benefit more as compared to the high-risk patients from ICB therapy may need more evidence. In addition, low-risk patients had heightened sensitivity to chemotherapy and targeted drugs. The GSEA results demonstrated that the drug-resistant pathways, including focal adhesion and extracellular matrix (ECM) receptor interaction, were upregulated in the high-risk group. Targeting of adhesion proteins can alter the behavior of cancer cells toward a more drug-sensitive phenotype of both chemotherapy and molecular therapeutics (34). Furthermore, HCC cells develop resistance to vandetanib (inhibitors of vascular endothelial growth factor) by adhering to the basement membrane component laminin-5 (35). The ECM receptor interaction was reported to be involved in multiple tumor progression (36, 37). When the stiffness of ECM changes, it can act as an obstacle to prevent the uptake and spread of drugs in the local environment of the cancer cells and further the influence drug response in HCC (38). The upregulation of the focal adhesion and ECM receptor interaction pathways may contribute to the elevated drug resistance in the high-risk group.

Despite the application value of the predictive model as described above, several limitations are present. The predictive model was constructed based on the data from public databases and the findings need to be rigorously assessed in a large patient cohort. Additionally, the finding on the prediction of therapeutic response should be validated in a clinical trial.

## Conclusions

In summary, we elucidated the prognostic roles of MED8 and characterized its pathophysiological significance in HCC, both of which have not been previously reported. Additionally, a predictive model was constructed based on a panel of three MED8-related immunomodulators. It was verified to have good power in discriminating the differences in therapeutic responses and it could supplement the prediction performance of TNM staging system in predicting HCC patients. This predictive model may have implications in prognosis stratification and therapeutic guidance for HCC.

## Data Availability Statement

The datasets presented in this study can be found in online repositories. The names of the repository/repositories and accession number(s) can be found in the article/**Supplementary Material**.

## Ethics Statement

The studies involving human participants were reviewed and approved by the Ethics Committee of the Taizhou Center Hospital (Taizhou University Affiliated Hospital). The patients/participants provided their written informed consent to participate in this study.

## Author Contributions

XJ, YS and JL designed the study. XJ and ZA performed the experiments. XJ, SW, DC, YF, CZ, LC, WT, ZZ, HL and JL analyzed the data. XJ drafted the manuscript. All authors contributed to the article and approved the final version.

## Funding

This work was supported by the National Natural Science Foundation of China (81870255), Zhejiang Provincial Science Foundation of China (LY21H020001, LQQ20H160001), Zhejiang Provincial Medicine & Healthcare technology project of China (2021KY306) and Ningbo Municipal Science Foundation of China (2021J296).

## Conflict of Interest

The authors declare that the research was conducted in the absence of any commercial or financial relationships that could be construed as a potential conflict of interest.

## Publisher’s Note

All claims expressed in this article are solely those of the authors and do not necessarily represent those of their affiliated organizations, or those of the publisher, the editors and the reviewers. Any product that may be evaluated in this article, or claim that may be made by its manufacturer, is not guaranteed or endorsed by the publisher.
