# Supplementary figures and images for: A Predictive Model for Prognosis and Therapeutic Response in Hepatocellular Carcinoma Based on a Panel of Three MED8-Related Immunomodulators
Source: Front Oncol. 2022 Apr 26;12:868411. doi: 10.3389/fonc.2022.868411 (PMC9086905; doi:10.3389/fonc.2022.868411)

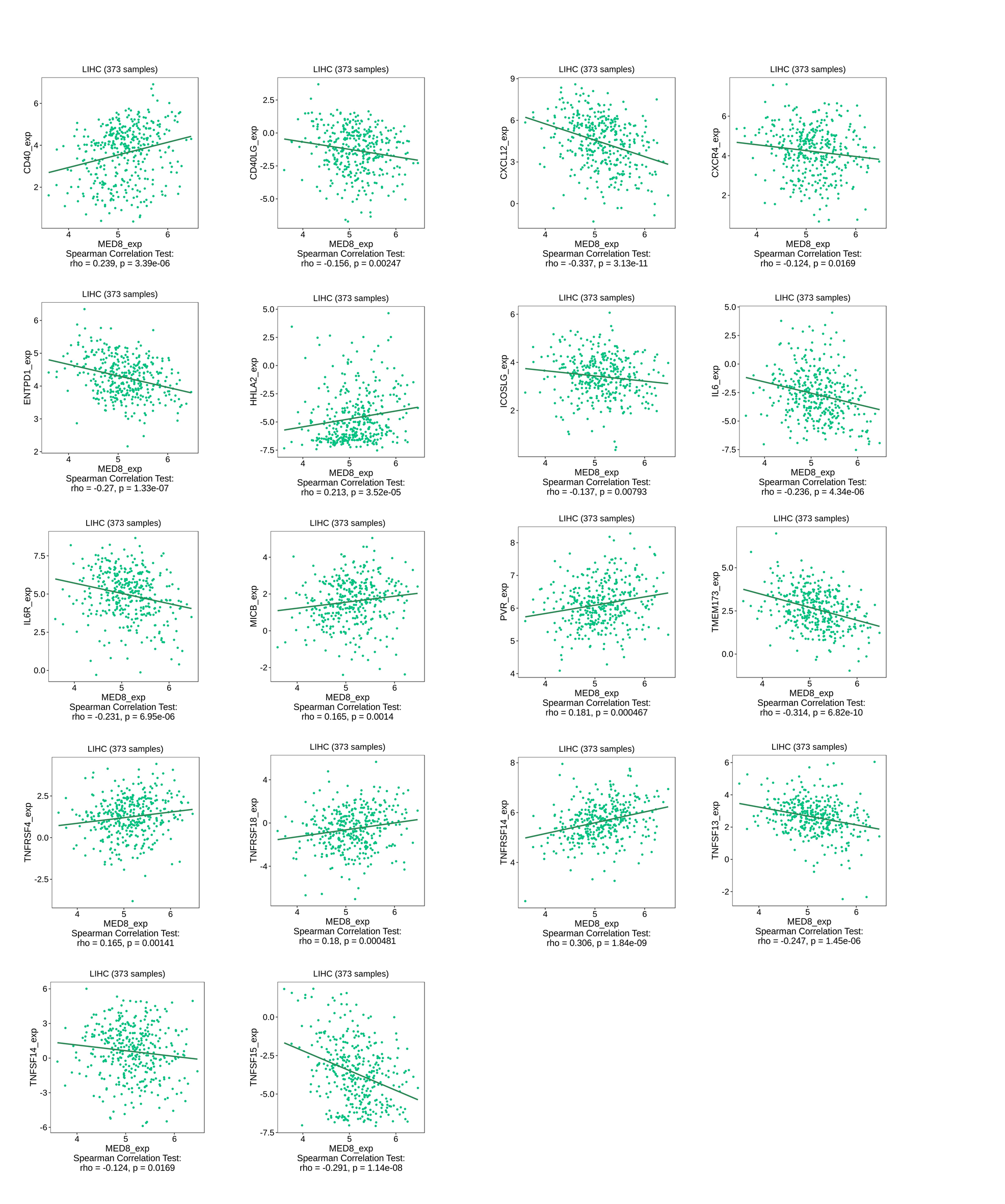

Supplement: Supplementary file 1 [file Image_1.tif]

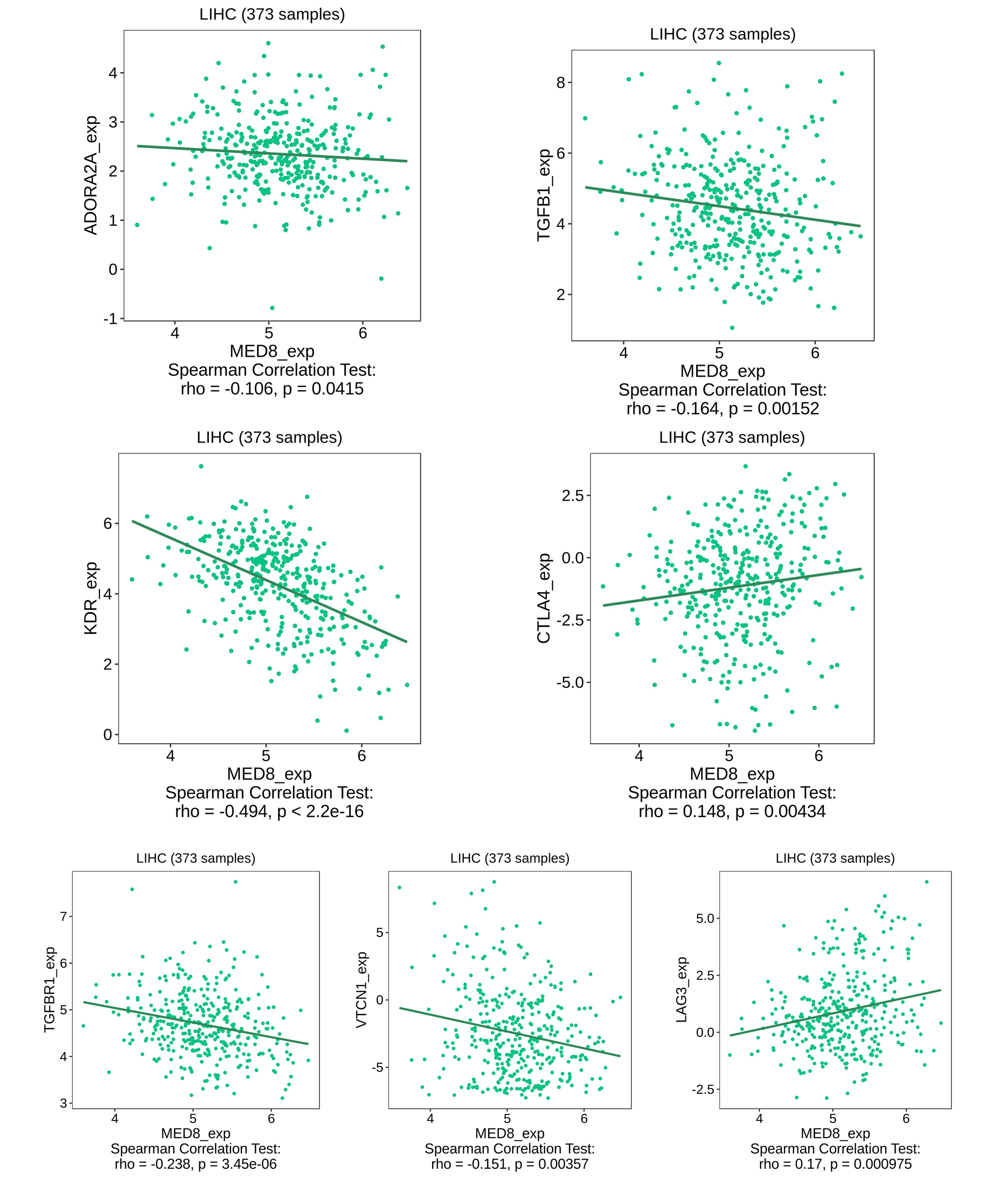

Supplement: Supplementary file 2 [file Image_2.tif]
