# Supplementary material for: A Predictive Model for Prognosis and Therapeutic Response in Hepatocellular Carcinoma Based on a Panel of Three MED8-Related Immunomodulators
Source: Front Oncol. 2022 Apr 26;12:868411. doi: 10.3389/fonc.2022.868411 (PMC9086905; doi:10.3389/fonc.2022.868411)
Supplement: Supplementary file 3 [file Table_1.docx]

Supplementary Table S1. The clinical features of HCC patients from TCGA and ICGC databases.

| Variables | Groups | TCGA-LIHC cohort (*n*=365) | ICGC cohort (*n*=240) |
| --- | --- | --- | --- |
| Survival status | Alive | 239 | 197 |
|  | dead | 126 | 43 |
| T stage | T1 | 180 |  |
|  | T2 | 91 |  |
|  | T3 | 78 |  |
|  | T4 | 13 |  |
|  | NA | 3 |  |
| N stage | N0 | 248 |  |
|  | N1 | 4 |  |
|  | NA | 113 |  |
| M stage | M0 | 263 |  |
|  | M1 | 3 |  |
|  | NA | 99 |  |
| Pathological stage | Stage1 | 170 | 36 |
|  | Stage2 | 84 | 109 |
|  | Stage3 | 83 | 74 |
|  | Stage4 | 4 | 21 |
|  | NA | 24 |  |
| Histological grade | Grade1 | 55 |  |
|  | Grade2 | 175 |  |
|  | Grade3 | 118 |  |
|  | Grade4 | 12 |  |
|  | NA | 5 |  |

NA: Not available.
