# Supplementary material for: A Predictive Model for Prognosis and Therapeutic Response in Hepatocellular Carcinoma Based on a Panel of Three MED8-Related Immunomodulators
Source: Front Oncol. 2022 Apr 26;12:868411. doi: 10.3389/fonc.2022.868411 (PMC9086905; doi:10.3389/fonc.2022.868411)
Supplement: Supplementary file 4 [file Table_2.docx]

Supplementary Table S2: The reference of the specific siRNA for human MED8.

| Targeted gene | References | |
| --- | --- | --- |
| Human mediator complex subunit 8 | Sense (5’-3’) | Antisense (5’-3’) |
|  | GGGAGUUUCAUUUGCAAGUTT | ACUUGCAAAUGAAACUCCCTT |

siRNA: Small interfering RNA.
