# Supplementary material for: A Predictive Model for Prognosis and Therapeutic Response in Hepatocellular Carcinoma Based on a Panel of Three MED8-Related Immunomodulators
Source: Front Oncol. 2022 Apr 26;12:868411. doi: 10.3389/fonc.2022.868411 (PMC9086905; doi:10.3389/fonc.2022.868411)
Supplement: Supplementary file 5 [file Table_3.docx]

Supplementary Table S3: The primer sequences.

| Gene | References | |
| --- | --- | --- |
| Human mediator complex subunit 8 | Forward (5’-3’) | Reverse (5’-3’) |
|  | ATTGGAGACCTTCAGGCAGC | ATGCATGGAAGCCGACTTGA |
