# Supplementary material for: A Predictive Model for Prognosis and Therapeutic Response in Hepatocellular Carcinoma Based on a Panel of Three MED8-Related Immunomodulators
Source: Front Oncol. 2022 Apr 26;12:868411. doi: 10.3389/fonc.2022.868411 (PMC9086905; doi:10.3389/fonc.2022.868411)
Supplement: Supplementary file 6 [file Table_4.docx]

Supplementary Table S4: The information of antibody.

| Antibodies | Information | |
| --- | --- | --- |
| Human mediator complex subunit 8 | Molecular weight | 29KD |
|  | Brand | Affinity |
|  | Product code | DF4154 |
| Human proliferating cell nuclear antigen | Molecular weight | 36KD |
|  | Brande | Cell signaling technology |
|  | Product code | 13110 |
| Human glyceraldehyde-3-phosphate dehydrogenase | Molecular weight | 37KD |
|  | Brande | Santa Cruz biotechnology |
|  | Product code | sc-32233 |
